# Isolation of an Extensively Drug-Resistant Pseudomonas aeruginosa *exoS*^+^/O4 Strain Belonging to the “High-Risk” Clone ST654 and Coproducer of NDM-1 and the Novel VIM-80

**DOI:** 10.1128/spectrum.01439-22

**Published:** 2022-10-10

**Authors:** Andrés Opazo-Capurro, Felipe Morales-León, Christian Jerez, Jorge Olivares-Pacheco, Manuel Alcalde-Rico, Paulina González-Muñoz, Helia Bello-Toledo, Adriana Cardenas-Arias, Fernanda Esposito, Nilton Lincopán, Vijna Illesca, Gerardo González-Rocha

**Affiliations:** a Laboratorio de Antibióticos, Departamento de Microbiología, Facultad de Ciencias Biológicas, Universidad de Concepción, Concepción, Chile; b Millennium Nucleus for Collaborative Research on Bacterial Resistance, Santiago, Chile; c Departamento de Farmacia, Facultad de Farmacia, Universidad de Concepción, Concepción, Chile; d Department of Restorative School of Dentistry, University of Concepción, Concepción, Chile; e Grupo de Resistencia Antibacteriana en Bacterias Patógenas Ambientales GRABPA, Instituto de Biología, Pontificia Universidad Católica de Valparaíso, Valparaíso, Chile; f Departamento de Ciencias Biológicas y Químicas, Facultad de Medicina y Ciencia, Universidad San Sebastián, Concepción, Chile; g Department of Microbiology, Institute of Biomedical Sciences, University of São Paulo, São Paulo, Brazil; h Department of Clinical Analysis, School of Pharmacy, University of São Paulo, São Paulo, Brazil; i Hospital Hernán Henríquez Aravena, Clinical Laboratory, Temuco, Chile; Instituto de Higiene

**Keywords:** carbapenem-resistant *Pseudomonas aeruginosa*, high-risk clone, ST654, NDM-1, VIM-80

## Abstract

The aim of this study was to investigate the genomic features of an extensively drug-resistant (XDR) Pseudomonas aeruginosa isolate (P-469) emerging in Chile. Antibiotic susceptibility was determined by disk diffusion and “colistin agar” test. Whole-genome sequencing (WGS) was performed by the Illumina NextSeq 2000 platform, and epidemiologically and clinically relevant data (i.e., sequence-type, serotype, mobile genetic elements, virulome, resistome, plasmidome, prophages, and CRISPR-Cas systems) were retrieved using multiple bioinformatic tools. The P-469 strain displayed an XDR profile, remaining susceptible to colistin. Genomic analysis revealed that this isolate belonged to the “high-risk” clone ST654 (CC654), serotype O4, and genotype *exoS^+^*. Strikingly, two CRISPR-Cas systems, five intact prophages sequences, and a broad resistome that included *bla*_NDM-1_ and the novel *bla*_VIM-80_ carbapenemase genes were predicted. Our results revealed the genomic characteristics of P. aeruginosa belonging to the high-risk clone ST654/O4 coproducing NDM-1 and VIM-80 in Chile, supporting that genomic surveillance is necessary to track the emergence and spread of epidemiologically successful WHO’s critical priority pathogens in order to prevent their rapid dissemination.

## LETTER

Pseudomonas aeruginosa is a major concern in clinical settings, associated with complicated infections in critically ill patients. Commonly, P. aeruginosa is frequently involved in hospital-acquired infections (HAIs), with elevated mortality rates (1). In this context, carbapenems (i.e., imipenem and meropenem) are important antibiotics to treat these infections; however, carbapenem-resistant P. aeruginosa (CRPA) isolates have been emerging worldwide (2). Due to its relevance, CRPA has been classified as a “priority one” pathogen by the World Health Organization (WHO) (3).

Epidemiologically, high-risk clones of P. aeruginosa have been defined based on their dominance, virulence, and multidrug resistance, being grouped within sequence types (STs) ST111, ST175, ST233, ST235, ST244, ST277, ST298, ST308, ST357, and ST654 (1, 2). Accordingly, it has been proposed that virulence factors (i.e., type III secretion system [T3SS]) play a relevant role in the severity of infections caused by these lineages (1). Moreover, CRPA isolates are normally associated with these clones (1, 2), in which the number of carbapenemases-producing P. aeruginosa isolates has been increasing in recent years (2). Among these enzymes, metallo-β-lactamases (MBLs), such as VIM carbapenemases, are the most prevalent in CRPA (2). In Chile, P. aeruginosa corresponds with the main etiological agent associated with ventilator-associated pneumonia (VAP) (https://www.minsal.cl/wp-content/uploads/2020/08/INFORME-DE-VIGILANCIA-DE-IAAS-2018.pdf), with carbapenem resistance rates ranging between 47.5% and 38.6% (https://atlas-surveillance.com, accessed in January 2022). Here, we present the genomic features of a carbapenem- and extensively drug-resistant (XDR) P. aeruginosa strain (P-469) emerging in Chile.

P. aeruginosa P-469 was recovered from bronchial lavage fluid of a patient with acute pneumonia, who was hospitalized in a public hospital in 2018 in Temuco, southern Chile. Antimicrobial susceptibility testing was carried out by the disk diffusion method and by the colistin agar test, according to the Clinical and Laboratory Standards Institute (CLSI) guidelines (4). Furthermore, the colistin MIC was determined using the ComASP colistin kit (Liofilchem), following the manufacturer’s protocol.

Then, P-469 was subjected to whole-genome sequencing (WGS) utilizing the NextSeq 2000 Illumina platform (2 × 150-bp paired-end reads). *De novo* assembly was performed by the bioinformatic tools of the Pathosystems Resource Integration Center (PATRIC; https://www.patricbrc.org). Next, the assembled genome was subjected to species identification by the Similar Genome Finder tool in PATRIC v3.6.12 (https://www.patricbrc.org). Afterward, antibiotic resistance genes (ARGs), STs, virulence factors, and prophage sequences were identified by ResFinder v4.1 (https://cge.food.dtu.dk/services/ResFinder/), MLST v2.0 (https://cge.food.dtu.dk/services/MLST/), Virulence Factor Database (VFDB; http://www.mgc.ac.cn/VFs/main.htm), and PHASTER (https://phaster.ca), respectively. Moreover, the plasmidome was determined by RFPlasmid (http://klif.uu.nl/rfplasmid/), whereas P. aeruginosa serotyping and the presence of CRISPR-Cas elements were analyzed by PAst v1.0 (https://cge.food.dtu.dk/services/PAst/) and CRISPRCasTyper (https://crisprcastyper.crispr.dk), respectively. Moreover, we aimed to carry out conjugation experiments to determine whether the carbapenemase-encoding genes present in P-469 are transferable. For this, we used a standard protocol utilizing the sodium azide-resistant Escherichia coli J53 strain as recipient (5).

The draft genome of P. aeruginosa P-469 has 6,805,913 bp with 65.83% G+C content. The assembled genome comprises 148 contigs, with an *N*_50_ value of 177,733 bp. From WGS analysis, we confirmed the species identification. Moreover, P-469 was classified as ST654 and characterized as O-antigen serotype O4, with the *exoS^+^* (*exoU^−^*) T3SS genotype. Interestingly, it has been determined that P. aeruginosa isolates associated with this genotype (*exoS^+^*/*exoU^−^*) are predominant in patients with cystic fibrosis (CF) (6). In this sense, it is well-known that ExoS and ExoU contribute greatly to the pathogenesis of P. aeruginosa. Moreover, *exoS* and *exoU* genes are generally mutually exclusive (7). Accordingly, it has been established that the *exoS^+^*/*exoU^−^* genotype is associated with chronic infections and worse clinical outcomes than other genotypes (8). Interestingly, the presence of the O4 serotype differs from previous data since this clone is normally associated with the O11 serotype (1). In the case of the O4 serotype, it is associated with bacteremic P. aeruginosa strains (9).

Additionally, P-469 exhibited resistance to carbapenems (imipenem and meropenem), cephalosporins (cefepime and ceftazidime), quinolones (levofloxacin and ciprofloxacin), aminoglycosides (amikacin and gentamicin), and β-lactams/β-lactamases inhibitors (piperacillin-tazobactam and ceftazidime-avibactam), remaining susceptible to colistin (MIC, 2 μg/mL); therefore, it was classified as XDR (1).

**FIG 1:**
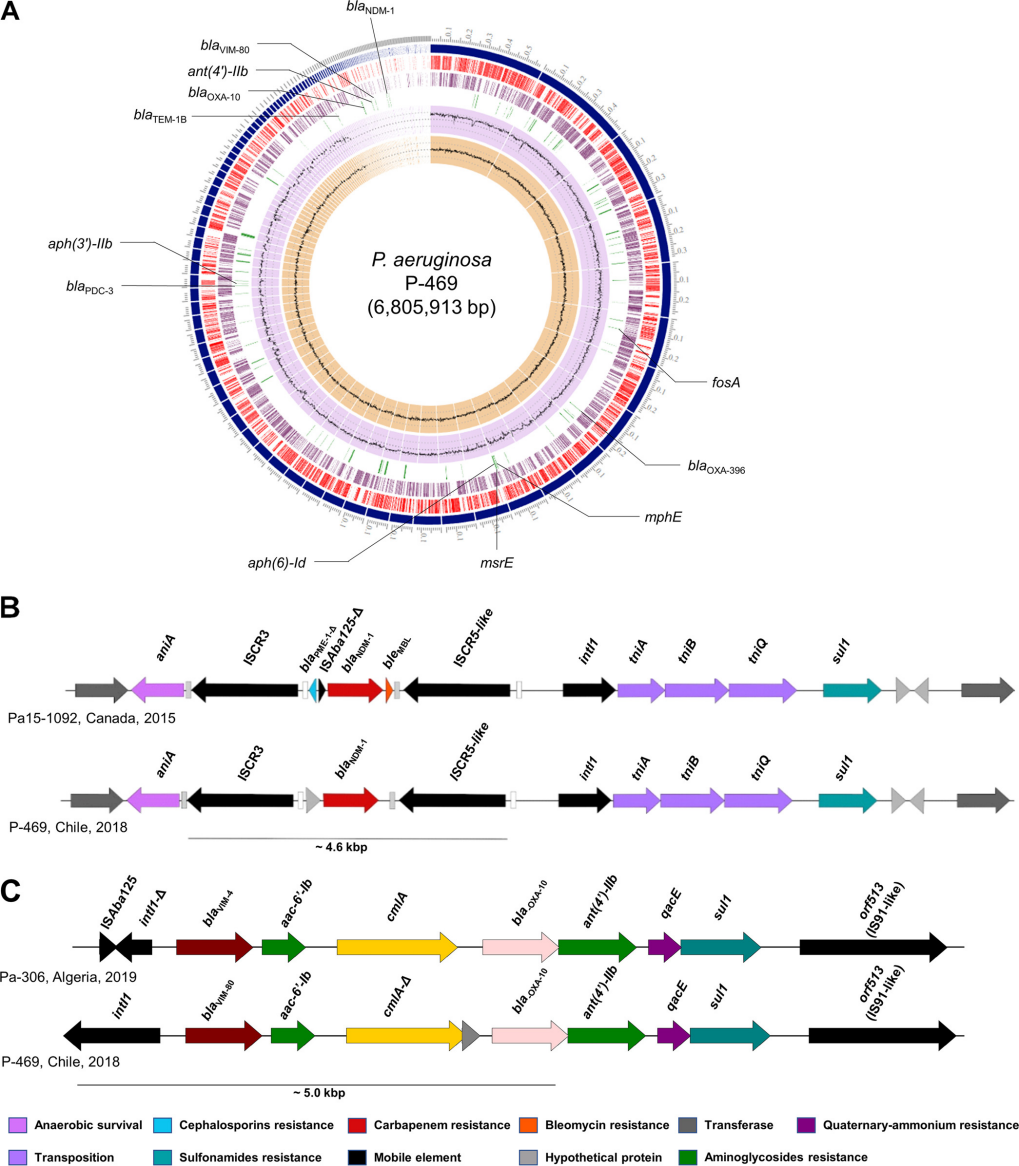
Circular genome map, comparison of the genetic context of the *bla*_NDM-1_ gene, and genetic context of the *bla*_VIM-80_ gene of clinical P. aeruginosa strain P-469. (A) Rings, beginning from the outermost ring, are as follows: contigs, forward coding DNA sequence (CDS), reverse CDS, acquired antibiotic resistance genes, GC content, and GC skew. (B) The *bla*_NDM-1_ gene is flanked by an ~4.6-kbp region composed of insertion sequence common region 3 (ISCR*3*)-*bla*_NDM-1_-ISCR*5*-like. The *oriIS* and *terIS* of ISCR elements are represented by light gray and white rectangles, respectively. (C) Genetic context of the *bla*_VIM-80_ gene.

The presence of several acquired ARGs was confirmed (Fig. 1A), including *bla*_PDC-3_, *bla*_TEM-1B_, *bla*_OXA-396_, *bla*_OXA-10_, *bla*_NDM-1_, and *bla*_VIM-_*_like_* (resistance to β-lactams), *mphE* and *msrE* (resistance to macrolides), *fosA* (fosfomycin), and *aph(*6*)-Id*, *aac(6′)-Ib*, *ant(4′)-IIb*, and *aph(3′)-IIb* (resistance to aminoglycosides), which is concordant with the observed phenotype. Interestingly, the amino acid sequence of the VIM-like metallo-β-lactamase presented a deletion at position 197 in comparison with VIM-2 (99.62% identity). This novel VIM variant was termed VIM-80 by the National Center for Biotechnology Information. Using the Protein Variation Effect Analyzer (PROVEAN) server (http://provean.jcvi.org), we determined that the deletion on position 197 in VIM-80 produces a deleterious effect on the protein in comparison to VIM-2. However, further analyses should be included to determine the effect of this mutation. Furthermore, we identified various intrinsic multidrug efflux pumps, such as MexAB-OprM, MuxABC-OpmB, MexXY-OprM, MexGHI-OpmD, MexVW-OprM, MexMN-OprM, MexJK, MexPQ-OpmE, and TriABC-OmpH systems, which can confer resistance to multiple antibiotics and biocides. From RFPlasmid results, 10 contigs (>1,000 bp) were predicted as plasmid associated and harbored *bla*_NDM-1_ and *bla*_OXA-10_ genes. Specifically, the *bla*_NDM-1_ was flanked by an ~4.6-kbp region composed of IS*CR3*-*bla*_NDM-1_-IS*CR5-*like (Fig. 1B), as previously described (1). However, P-469 did not contain the truncated *bla*_PME-1_ and *ble*_MBL_, which were present in the Pa-1092 genome (GenBank accession number CP012901). On the other hand, the genetic context of the novel *bla*_VIM-80_ variant was associated with a class 1 integron (Fig. 1C), which is similar to the structure identified in a VIM-4-producing P. aeruginosa isolate collected in Algeria in 2019. Furthermore, the P-469 strain contains two CRISPR-Cas systems that belong to the I-F and I-E subtypes, and it harbored five intact prophages sequences. Conjugation was not achieved under the experimental conditions. To determine the genetic context of both carbapenemases with more resolution, complementing Illumina data with long-read sequencing (i.e., Oxford Nanopore Technologies) can be performed in the future and will reveal whether they are plasmid located.

In summary, our results provide relevant genomic data on P. aeruginosa belonging to the high-risk clone ST654/O4, coproducing NDM-1 and VIM-80, emerging in Chile, establishing a baseline for future genomic studies on the epidemiology of this high-risk WHO critical priority clone in Latin America.

### Data availability.

This whole-genome shotgun project has been deposited at DDBJ/ENA/GenBank under accession no. JACTAE000000000. The version described in this paper is version JACTAE000000000.1. The novel VIM variant was deposited at the National Center for Biotechnology Information under accession number MBC9045152.
